# Autoimmune thyroid disease with myasthenia gravis in a 28-year-old male: a case report

**DOI:** 10.4076/1757-1626-2-8766

**Published:** 2009-09-14

**Authors:** Imran Masood, Mir Yasir, Aiffa Aiman, R P Kudyar

**Affiliations:** 1 Department of Medicine, ASCOMS & Hospitals, Sidhra, Jammu, Jammu & Kashmir, 180017, India; 2 Department of Surgery, ASCOMS & Hospitals, Sidhra, Jammu, Jammu & Kashmir, 180017, India; 3 Department of Pathology, GMC, Bakshinagar, Jammu, Jammu & Kashmir, 180017, India

## Abstract

**Introduction:**

Graves' disease and myasthenia gravis are both auto-immune diseases and the coexistence of these two diseases is rare but well recognized. Myasthenia gravis is more frequent in patients with thyroid disease.

**Case presentation:**

Here we present a case of 28-year-old male patient having Auto-immune thyroid disease (Graves' disease) with concomitant myasthenia gravis.

**Conclusion:**

In conclusion, we report that the coexistence of Myasthenia Gravis with Autoimmune thyroid disease might have prognostic relevance and occurs in a subgroup of myasthenia gravis patients with a mild form of the disease.

## Introduction

Robert Graves' disease, although first described by Parry in 1825 [1], is best known as Graves' disease and is characterized by diffuse goiter (Thyrotoxicosis), infiltrative orbitopathy & ophthalmopathy and occasionally infiltrative dermopathy. It occurs in up to 2% of women and 0.2% in men. The disorder rarely begins before adolescence and typically occurs between 20 and 50 years of age and rarely it occurs in elderly as well. It is an autoimmune disorder with autoantibodies directed against the TSHR (TSHR Abs) and behave as thyroid-stimulating antibodies [2,3]. Pathologically Graves' disease, is characterized by a non-homogeneous lymphocytic (T-cells: Th1 and Th2 types along with CD25+ regulatory T cells; while B-cell germinal centers are much less common) infiltration with an absence of follicular destruction [4]. Clinically it is characterized by hyperactivity, irritability, dysphoria, heat intolerance, sweating, palpitations, fatigue, weakness, weight loss despite increased appetite, diarrhea, polyuria, oligomenorrhea, loss of libido, tachycardia, atrial fibrillation, tremors, goiter, warm, moist skin, muscle weakness, proximal myopathy, lid retraction/lag, gynecomastia [5]. Other auto-immune disorders occurring in association with Graves' disease include Acquired hypokalemic periodic paralysis, Myasthenia gravis, Insulin-dependent diabetes mellitus, Pernicious anemia, Adrenal atrophy, Sjogren's syndrome, Lupus erythematosus, Rheumatoid arthritis and Idiopathic thrombocytopenic purpura, vitiligo.

## Case presentation

A 28-year-old Indian male, with Indoaryan ethnicity non-smoker, non-alcoholic patient, laborer by occupation presented to hospital with a history of gradually increasing painful swelling in the neck of 3 months duration associated with difficulty in swallowing and palpitations. It was not associated with hoarseness of voice, difficulty in breathing. Patient also complained of low grade fever of 3 months duration, continuous, associated with chills but no rigors or any diurnal variations. There was also history of generalized weakness, diplopia on downward gaze, drooping of eyelids on both sides. Patient had no such complaint in past or no such history was found in family or first degree relatives. On examination patient was conscious, oriented, with nasal twang speech. Look was lethargic and had proptosis and ptosis. Large thyroid swelling was visible, bilaterally symmetrical and tender, smooth, firm in consistency, non-fluctuant, with audible bruit present. B.P-100/60 mmHg, Pulse- 98/min, Temp- 100 0F and respiratory rate of 18/min. Examination also revealed bilateral weakness of superior recti, inferior oblique, lateral recti and left medial rectus.

**Figure 1:**
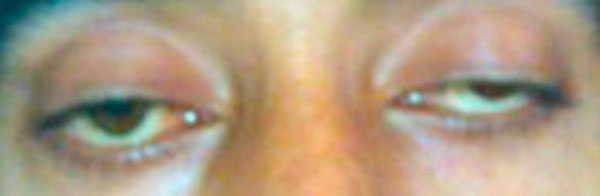
**Showing ptosis before neostigmine test**.

**Figure 2:**
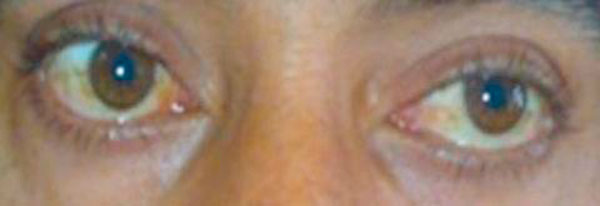
**Showing improvement in ptosis after neostigmine test**.

Investigations revealed Hb-12 g/dl, TLC-9,000/cmm, DLC- N68 E3 L27 M2 B0, Platelets - 2.25 lacs/cmm, ESR - 35 mm, P.B.F - Normocytic normochromic picture, Urea-13 mg/dL, Bl. Glucose (R)- 92 mg/dL, Sr. Creatinine-0.5 mg/dL, Sr. total protein- 6.9 g/dL, Sr. albumin- 3.1, SGOT- 56 U/L, SGPT -20 U/L, ALP- 116 U/L, Sr.Ca++= 1.01 mmol/L, Phosphate-4.0 mg/dl, S.sodium-136 meq/l, Sr. K+-2.9 meq/l, Chest X-ray-Normal, X-ray neck- soft tissue swelling anterior to trachea, routine urine exam- normal, ECG- PR interval prolonged, flattened T waves, TSH- 0.005 µIU/ml, Free T4- 6.89 ng/dL, Free T3- 16.19 pg/ml. FNAC thyroid- thyroid follicular cells, colloid, Hurthle cells and lymphocytes (features suggestive of Hashimoto's thyroiditis), and Neostigmine test for myasthenia gravis- Positive (Figures 1 and 2).

In addition to Neomercazole 10 mg three times daily, Pyridostigmine 30 mg three times daily was added for the treatment of ptosis and increased to 60 mg t.i.d., with patient showing dramatic improvement both in symptoms and signs after the start of therapy.

## Discussion

Patients with myasthenia gravis may have coexisting AITD, which include Hashimoto's disease Type 2A and Graves' disease Type 3A. Epidemiological studies show that AITD occur in approximately 5-10% of patients with MG, whereas MG is reported in a fairly low frequency (0.2%) of patients with AITD. The clinical presentation of MG associated with AITD is frequently restricted to eye muscles. The reason for the association of AITD with ocular MG is unknown, but several hypotheses can be considered. First, ocular MG and generalized MG might actually represent separate diseases with different spectra of associated conditions. Second, an immunological cross-reactivity against epitopes or auto-antigens shared by the thyroid and the eye muscles might be the basis of this association. A third explanation for the higher frequency of ocular MG in AITD could be that these disorders have a common genetic background [6]. In three-quarters of patients with both conditions, thyrotoxic symptoms occur before or concurrently with those of myasthenia [7]. Our patient had thyrotoxicosis symptoms 2 months before his ptosis developed. The ocular changes in Graves' disease may include exophthalmos, periorbital edema, lid lag, chemosis and ophthalmoplegia. The extraocular muscles most commonly involved in Graves' disease are the superior and lateral recti [8]. Two-thirds of the patients with both disorders show improvement in myasthenia gravis after treatment of thyroid disease [8].

## Conclusion

In conclusion, we report that the coexistence of MG with AITD might have prognostic relevance and occurs in a subgroup of MG patients with a mild form of the disease. It should be remembered that, ptosis is not an expected symptom in thyroid ophthalmopathy. If ptosis or paresis of the orbicularis oculi muscle develops in a patient with thyroid ophthalmopathy, coincidence of myasthenia gravis should be considered.

## Consent

Written informed consent was obtained from the patient for publication of this report and accompanying images. A copy of written consent is available for review by Editor-in-Chief of this journal.

## Competing interests

The authors declare that they have no competing interests.

## Author's contributions

IM gave conception, design and helped in acquisition of data. MY interacted with the patient and his family and helped in analysis and interpretation of data. AA helped in performing all the necessary investigations and in revising the patient data. RPK guided in interpretation of case and approval of the final manuscript.

## References

[B1] Parry CH Enlargement of the Thyroid Gland in connection with Enlargement or Palpitation of the Heart 1825 Underwoods Fleet-Street 111 129

[B2] Davies TF Ando T Lin RY Tomer Y Latif R Thyrotropin receptor-associated diseases: from adenomata to Graves disease J Clin Invest 2005 115 1972 1983 10.1172/JCI26031 16075037 PMC1180562

[B3] Rees Smith B McLachlan SM Furmaniak J Autoantibodies to the thyrotropin receptor Endocr Rev 1988 9 106 121 10.1210/edrv-9-1-106 3286231

[B4] Weetman AP Cellular immune responses in autoimmune thyroid disease Clin Endocrinol 2004 61 405 10.1111/j.1365-2265.2004.02085.x 15473869

[B5] Klein I Ojamaa K Thyroid hormone and the cardiovascular system N Engl J Med 2001 344 501 10.1056/NEJM200102153440707 11172193

[B6] Marino M Ricciardi R Pinchera A Barbesino G Manetti L Chiovato L Braverman LE Rossi B Muratorio A Mariotti S Mild Clinical Expression of Myasthenia Gravis Associated With Auto-immune Thyroid Diseases J Clin Endocrinol Metab 1997 82 438 443 10.1210/jc.82.2.438 9024233

[B7] Bartley GB Epidemiologic characteristics and clinical course of ophthalmopathy associated with auto-immune thyroid disease in Olmsted County. Minnesota Trans Am Ophithalmol Soc 1994 92 477 588 PMC1298522 7886878

[B8] Ali AS Akavaram NR Neuromuscular disorders in thyrotoxicosis Am Fam Physician 1980 22 97 102 6893387

